# Hepatocellular Carcinoma in Non-Alcoholic Fatty Liver Disease: From Epidemiology to Diagnostic Approach

**DOI:** 10.3390/cancers13225844

**Published:** 2021-11-21

**Authors:** Ivica Grgurevic, Tonci Bozin, Mislav Mikus, Michal Kukla, James O’Beirne

**Affiliations:** 1 Department of Gastroenterology, Hepatology and Clinical Nutrition, University Hospital Dubrava, 10 000 Zagreb, Croatia; tbozin@kbd.hr; 2 Faculty of Pharmacy and Biochemistry, School of Medicine, University of Zagreb, 10 000 Zagreb, Croatia; 3 Department of Obstetrics and Gynecology, University Hospital Centre Zagreb, 10 000 Zagreb, Croatia; mmikus@mef.hr; 4 Department of Internal Medicine and Geriatrics, Faculty of Medicine, Jagiellonian University Medical College, 30688 Cracow, Poland; michal.kukla@uj.edu.pl; 5 Department of Hepatology, University of the Sunshine Coast, Sunshine Coast 4556, Australia; jobeirne@usc.edu.au

**Keywords:** hepatocellular carcinoma, non-alcoholic fatty liver disease, metabolic syndrome, diabetes mellitus, screening programs, biomarkers, ultrasound

## Abstract

**Simple Summary:**

Non-alcoholic fatty liver disease (NAFLD) is expected to become the leading cause of hepatocellular carcinoma (HCC) in the near future. In this article, we review the current knowledge about the epidemiology, risk factors, pathogenesis, clinical presentation and diagnostic approach to HCC in NAFLD. Knowledge of these facts is of great importance to improve the early identification of patients that are at risk, allowing for early detection of HCC and, thus, an improvement in clinical outcomes. This is especially important given that around 30% of NAFLD-related HCCs develop in a non-cirrhotic liver. The presence of diabetes, male sex, older age and Hispanic race, in addition to liver cirrhosis, are the most important risk factors for HCC in this setting. In summarising the current knowledge of genetic susceptibility, metabolic derangements and immunological mechanisms involved in the pathogenesis of NAFLD-related HCC, we illustrate the need for further research on this intriguing topic.

**Abstract:**

Non-alcoholic fatty liver disease (NAFLD) is becoming the leading cause of liver morbidity worldwide and, as such, represents the pathogenic background for the increasing incidence of hepatocellular carcinoma (HCC). The annual incidence of NAFLD-related HCC is expected to increase by 45–130% by 2030. Diabetes mellitus is the most important risk factor for HCC development in NAFLD, with the risk further increased when associated with other metabolic traits, such as obesity, arterial hypertension and dyslipidemia. The highest risk of HCC exists in patients with advanced fibrosis or cirrhosis, although 20–50% of HCC cases arise in NAFLD patients with an absence of cirrhosis. This calls for further investigation of the pathogenic mechanisms that are involved in hepatocarcinogenesis, including genetics, metabolomics, the influence of the gut microbiota and immunological responses. Early identification of patients with or at risk of NAFLD is of utmost importance to improve outcomes. As NAFLD is highly prevalent in the community, the identification of cases should rely upon simple demographic and clinical characteristics. Once identified, these patients should then be evaluated for the presence of advanced fibrosis or cirrhosis and subsequently enter HCC surveillance programs if appropriate. A significant problem is the early recognition of non-cirrhotic NAFLD patients who will develop HCC, where new biomarkers and scores are potential solutions to tackle this issue.

## 1. Introduction

Primary liver cancer is the sixth most frequently diagnosed cancer in the world and holds third place in terms of global cancer mortality according to the 2020 GLOBOCAN report [1,2]. The most prevalent histological type of primary liver cancer is hepatocellular carcinoma (HCC), accounting for 75–85% of all cases, followed by intrahepatic cholangiocarcinoma and other rare types of tumors; thus, the reported epidemiological data mostly reflects the situation with HCC. The incidence of newly diagnosed cases and the number of deaths per year are almost the same, indicating poor prognosis and significant gaps that exist in identifying and treating patients with HCC [1,2]. The risk factors for HCC development are multiple, but the best recognized are chronic viral hepatitis B and C; alcohol-related chronic liver disease; obesity and metabolic syndrome as the underlying causes for non-alcoholic fatty liver disease (NAFLD); some dietary factors, such as high iron intake; aflatoxin; cigarette smoking; and genetic susceptibility [1]. HCC develops on the background of liver cirrhosis in more than 85% of cases [1]. This association has been recognized for years and forms the basis for clinical practice guidelines that recommend efforts to identify patients with cirrhosis and then to survey them every 6 months using a liver ultrasound (US) with/without alpha-fetoprotein (AFP) in order to detect HCC at an early treatable stage [3,4,5]. In the remaining 15% of patients with HCC who do not have cirrhosis, HCC most usually arises in the context of chronic hepatitis B and NAFLD. HCC incidence rates increase with age and are higher in men and among some ethnic/racial groups, with the highest age-standardized incidence rates reported in Eastern Asia, Northern Africa and the Pacific Islands [1,2]. Poor prognosis, unrecognized steps in pathogenesis and challenging issues in diagnosing HCC, especially in the absence of cirrhosis, call for further research in resolving these open questions. This is particularly important due to the epidemics of overweight/obesity and NAFLD that are becoming the leading causes of HCC [6]. This review summarizes the current knowledge that is related to the epidemiology, pathogenesis and diagnostic approaches to HCC in patients with NAFLD.

## 2. Epidemiology

Chronic liver disease (CLD) represents a significant burden for health systems, with alcoholic liver disease (ALD) and chronic viral hepatitis B and C (CHB and CHC) being traditionally considered the most prevalent causes of liver cirrhosis and HCC. Indeed, alcohol consumption still represents a major public health issue and Europe is the region with the highest yearly per capita alcohol consumption in the world [7]. On the other hand, vaccination against CHB and effective treatment of CHC using direct-acting antiviral (DAA) drugs have resulted in a decreasing prevalence of viral hepatitis worldwide. Despite the Global Health Sector Strategy on Viral Hepatitis 2016–2021 being launched by the World Health Organization, which aims at reducing new HCV infections by 90% and reducing deaths due to viral hepatitis by 65% by 2030, these goals will probably not be reached by the defined deadlines, and the current situation with the COVID-19 pandemic further compromises the achievement of these goals [8,9]. In the meantime, obesity, which is another global pandemic, has also been well recognized as a significant global health threat, not only for cardiovascular morbidity and mortality but also as an important cause of chronic liver disease (CLD) [10,11]. NAFLD due to overweight/obesity, in association with type 2 diabetes, arterial hypertension and dyslipidemia, is by far the most important etiology of this silent pandemic, for which reason, some authors proposed an alternative term: metabolic-associated fatty liver disease (MAFLD) [11,12]. NAFLD affects around one-quarter of the adult population, especially in developed countries, where its prevalence correlates with that of obesity [11,12]. Liver-related mortality, including that due to HCC, is the third most common cause of death amongst patients with NAFLD, with cardiovascular causes and extrahepatic malignancy being the first two causes [13]. The risk for both overall and liver-related mortality increases with the incremental worsening of liver fibrosis stages, with the most significant increase in the prevalence of these complications being observed at the stage of advanced (F3) fibrosis [14]. Progressive fibrosis develops in around one-third of patients, whereas cirrhosis eventually develops in 5–10% of all patients with NAFLD [15,16]. Cirrhosis as the consequence of NAFLD is becoming the leading cause of liver transplantation in many regions of the world [17,18].

According to the results from a large multiregional study (BRIDGE study) conducted over the period 2012–2015 that included 42 sites in North America, Europe and the Asia–Pacific region, with data collected from 18,031 HCC patients, NAFLD was the fourth most common etiology of HCC (after viral hepatitis B and C and ALD), with 12 and 10% of cases in North America and Europe, respectively, being attributable to this etiology (Table 1). In the Asia–Pacific region, the prevalence of NAFLD as a cause of HCC was lower, ranging from 1 to 6% [19]. Corresponding figures for African countries were 12% for Egypt and 22% for other countries. Of note, an assumed diagnosis of NAFLD as the underlying etiology for HCC in this study relied upon the exclusion of hepatitis B and C and alcohol-related liver disease. The highest reported proportion of NAFLD as the background etiology of HCC was 58% according to a study from the United States of America (USA) [20].

However, temporal trends over the last two decades reveal a steady increase in the prevalence of NAFLD as the underlying liver disease in patients that are diagnosed with HCC. For example, according to the Scientific Registry of Transplant Recipients in the USA, NAFLD-related HCC prevalence was 2.1% in 2002 and rose to 16.2% in 2016 [21]. A similar trend was observed in the UK where <10% of cases were attributable to NAFLD in 2000, as opposed to 34.8% in 2010, as well as in France, where 2.6 and 19.5% of HCC cases were reported to have developed due to NAFLD in the periods of 1995–1999 and 2010–2014, respectively [22,23]. The estimated annual incidence and prevalence of HCC related to NAFLD are expected to increase further by 44–122% and 47–120%, respectively, by 2030 [3,20,25].

Therefore, it is important to understand the risk factors that are associated with the progression from liver steatosis to HCC. In a study from the USA that was conducted involving almost 300,000 NAFLD patients and the same number of age- and sex-matched controls from the general population, after 9 years of follow up, the incidence rates of HCC were 10 times higher amongst the NAFLD patients compared to the age- and sex-matched controls (0.21 vs. 0.02/1000 person-years (PY)) [26]. In a multivariable analysis, the risk of HCC remained 7.62 times higher in NAFLD after adjustments for race and metabolic profile. Amongst patients with NAFLD, incidence rates varied significantly according to age, sex, race and metabolic conditions, but the strongest risk factor was the presence of cirrhosis (incidence rate 10.6/1000 PY). Nevertheless, in 20% of cases, HCC developed in the absence of cirrhosis. The incidence rate of HCC among NAFLD patients without cirrhosis was 0.08 per 1000 PY, which was comparable to the patients with diabetes but without NAFLD (0.07/1000 PY). Amongst patients with NAFLD cirrhosis, the highest risk of HCC was observed among older (>65 years of age) Hispanics (incidence rate 23.7/1000 PY), whereas it was 12.3/1000 PY in patients suffering from diabetes. This cohort was additionally analyzed in another study that focused on the association between metabolic traits and the development of HCC [24]. Each additional metabolic trait added to the risk of developing cirrhosis or HCC, where the strongest relative influence was from diabetes. Indeed, the concomitant presence of diabetes in patients with obesity and hypertension substantially increased the risk of developing HCC when compared with patients without diabetes (HR 8.63 and 1.07 respectively). The presence of diabetes was independently associated with a 2.8-fold higher risk of progressing to HCC in the overall cohort and a twofold higher risk among the patients who developed HCC in the absence of cirrhosis. In another study from the Mayo Clinic, the presence of diabetes increased the risk of HCC in NAFLD-related cirrhosis by fourfold (HR 4.2, 95% CI 1.2–14.2; *p* = 0.02) [27]. According to a systematic review and meta-analysis, the prevalence of NAFLD-related HCC that arises in non-cirrhotic liver was 38% as opposed to 14% in other etiologies of CLD [28] (Table 1). The reported incidence of HCC arising in non-cirrhotic liver was 0.57/1000 PY (NAFLD) and 1.32/1000 PY (NASH) in Spain, 0.37/1000 PY (NAFLD or NASH) in the Netherlands, 0.29/1000 PY (NAFLD or NASH) in Italy, 0.08/1000 PY in the USA and 0.9/1000 PY in Hong Kong. The reported annual incidence in Japan ranged from 0.043 to 0.4%, whereas the 10-year cumulative incidence was around 6% in Japan and 2.73% in Taiwan [3]. Similarly, a multicentric study from the USA demonstrated a fivefold higher incidence of HCC amongst non-cirrhotic NAFLD patients as compared with patients with non-cirrhotic chronic hepatitis C [29].

Although the relative risk is clearly increased in NAFLD patients, the absolute risk still remains low, with a 5- and 10-year cumulative incidence of 0.8 and 1.7 HCC cases per 1000 patients, respectively, according to a study from the USA [26]. Therefore, the authors concluded that the incidence of HCC among NAFLD patients without cirrhosis was still below the accepted thresholds (estimated HCC annual incidence 0.8–2.3%) that are considered relevant in terms of cost-effectiveness to initiate surveillance programs. Other studies have reported annual incidences of HCC in NAFLD cirrhosis ranging from 0.7 to 2.6% [3,30].

To conclude, NAFLD is becoming the leading cause of cirrhosis and HCC worldwide. Older age, race and the presence of diabetes represent the most important risk factors for the progression of NAFLD to HCC, and this may occur in a significant proportion of patients, even in the absence of cirrhosis. Therefore, to better stratify the risk of HCC, especially in non-cirrhotic NAFLD, it is important to understand the pathogenesis and identify the risk factors that are associated with this unfavorable development.

## 3. Pathogenesis

### 3.1. Genetic Background

Genetic predisposition plays an important role in the susceptibility to NAFLD and with the development of NAFLD-related HCC. To date, several differential gene expression mechanisms that result from various single-gene mutations and/or genetic instability, epigenetic changes and microRNA (miRNA)-altered expression have been reported (Table 2).

Single nucleotide polymorphisms were found to be an important factor that accounts for the NAFLD-related development of HCC. Polymorphisms in two particular genes are more prevalent in patients with non-alcoholic steatohepatitis (NASH), patatin-like phospholipase domain-containing 3 (*PNPLA3*) and transmembrane 6 superfamily member 2 (*TM6SF2*). The *PNPLA3* variant rs738409 known as p.I148M is related to increased liver lipid accumulation in patients, predisposing them to NAFLD, NASH and HCC [31]. Furthermore, *PNPLA3* mutations were demonstrated to be a factor in HCC progression, as overexpression was shown in a mouse liver model with triglyceride accumulation, triglyceride hydrolysis impairment and increased free fatty acid synthesis [32]. The *TM6SF2* variant rs58542926 known as p.E167K was associated with steatosis and fibrosis in NASH patients, independently of diabetes mellitus (DM), obesity or *PNPLA3* variant [33,34]. Current evidence revealed this variant to be an initial factor in NASH-related pathogenesis, as it is associated with liver injury in NAFLD-related HCC pathogenesis, but there is no supporting data regarding its role in HCC progression [35]. Two more gene variants were shown to contribute to HCC development: hemochromatosis (HFE) *H63D* gene and the rs641738 genotype encoding membrane-bound O-acyltransferase domain-containing 7 (*MBOAT7*). *HFE H63D* polymorphism was found to be increased in non-cirrhotic NAFLD-related HCC, with consequent inflammation, fibrosis and carcinogenesis due to increased iron accumulation [36]. The MBOAT7 protein was associated with liver damage and fibrosis risk in NAFLD patients [37]. Similar to the *TM6SF2* variant, further research is needed to elucidate its role in HCC progression [38].

Genetic instability in NASH patients appears to be higher in comparison with NAFLD patients with bland steatosis, and it is considered to be a risk factor for NAFLD-related HCC. One proposed mechanism suggests DNA amplification in genes is involved in oncogenic mechanisms that encode proteins for tumor growth [39]. Moreover, HCC was shown to have the highest prevalence of mutations in oncogenic genes [40,41], as shown in Table 2.

Epigenetic alterations that are associated with NASH-related HCC were found to induce DNA methylation of the *CHD1* gene encoding chromodomain helicase DNA-binding protein 1 [42], which leads to gene silencing in DNA damage and repair, and is related to lipid metabolism and fibrosis progression [43].

The role of miRNA in the pathogenesis of NASH was analyzed by using a microassay encompassing 474 human microRNAs, and the authors reported the altered expression of 23 miRNAs between the patients with NASH and those with normal liver histology. The most prominent finding was the underexpression of miR-122 in NASH patients, suggesting an association with altered lipid metabolism that is implicated in NASH pathogenesis [44], Additionally, miR-122 was also demonstrated to have a direct role in a mouse model of NASH-related hepatocarcinogenesis [45].

To elucidate the genetic background of NAFLD, several genome-wide association studies (GWAS) and detailed candidate gene analyses were independently validated and conducted [46]. For instance, the aforementioned *PNPLA3* was consistently identified as a NAFLD pathogenesis modifier, independently of whether biochemical indices or radiologically determined TAG accumulation were used in the assessment [47,48,49]. The *PNPLA3* gene provides information for adiponutrin synthesis, which is mainly found in adipocytes and hepatocytes [48]. The molecular mechanisms of the role *PNPLA3* plays in the promotion of steatosis, fibrosis and liver carcinogenesis were reviewed by Trepo et al. [50]. One of the mechanisms demonstrated using recombinant human PNPLA3 and PNPLA3-I148M proteins in mice showed that I148M substitution remains an important factor in reducing fatty acid release, promoting TG accumulation in hepatocytes and reducing the associated loss of hepatic function [51]. Although the proposed mechanisms link the PNPLA3 protein with steatosis and, to a lesser extent, fibrogenesis, there is yet insufficient data on its role in HCC development [50].

The Pro446Leu (rs1260326) glucokinase regulator gene (*GCKR*) variant results in a persistent increase in glucose uptake by the liver [52]. Such hepatic glycolysis that is associated with Pro446Leu increases the production of intracellular malonyl-CoA by suppressing glucose and insulin levels [46]. Increased production of intracellular malonyl-CoA further results in mitochondrial FA β-oxidation impairment [46].

According to the given evidence, there is a strong association between *TM6SF2* and multiple aspects of metabolic-syndrome–related end-organ damage [53]. Specifically, *TM6SF2* rs58542926 T allele mediates hepatic TG and cholesterol retention, promoting hepatic steatosis and fibrosis.

Despite the growing evidence regarding the genetic background in NAFLD pathogenesis, additional GWAS will be required to identify new variants that are associated with liver damage and cancer to explain a greater proportion of the heritability of these phenotypes.

### 3.2. Metabolic Disbalance

**Figure 1:**
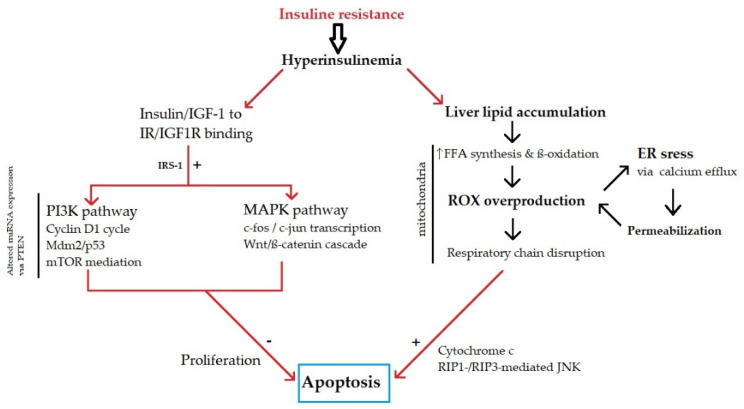
Metabolic pathogenesis of non-alcoholic fatty liver disease (NAFLD)-related hepatocellular carcinoma (HCC) (based on the references [45,46,47,48,49,50,51,52]). PI3K—phosphoinositide 3-kinase; MAPK—mitogen-activated protein kinase; IGF-1—insulin-like growth factor-1 (IGF-1); IRS-1—insulin receptor substrate 1; mTOR—mechanistic target of rapamycin; FFAs—free fatty acids; ER—endoplasmatic reticulum; PTEN—phosphatase and tensin homolog; RIP—receptor-interacting serine/threonine kinase; JNK—c-Jun N-terminal kinase.

Excessive consumption of food that is rich in saturated fat, refined carbohydrates, trans-fat, salt and sugar and the maintenance of a sedentary, modern lifestyle promote weight gain and obesity. During the weight gain process, other factors, such as altered gut microbiota, postprandial chronic inflammation and insulin/leptin resistance, can result in further accumulation of adipose tissue and increase the risk of chronic disease. The abovementioned metabolic factors are closely related to insulin resistance and hyperinsulinemia, which activate insulin receptor signaling via the phosphoinositide 3-kinase (PI3K) and mitogen-activated protein kinase (MAPK) pathways, resulting in metabolic disbalance and disrupted hepatocyte cell cycle control (Figure 1). First, insulin resistance and hyperinsulinemia, with insulin and insulin-like growth factor-1 (IGF-1) binding to the IR/IGF1R receptor, are triggered via the insulin receptor substrate 1 (IRS-1) signaling cascade through PI3K and MAPK pathways, with cell proliferation induction and apoptosis inhibition being main factors in HCC pathogenesis [54,55,56]. Furthermore, the MAPK pathway affects cell growth by inducing the transcription of the protooncogenes, c-Fos and c-Jun, and subsequent activation of the Wnt/beta-catenin signaling cascade. In the circumstances that are produced by such a metabolic microenvironment, fibrosis and carcinogenesis in the liver are promoted [56,57]. Moreover, altered miRNA expression in hepatocarcinogenic pathways (MAPK and PI3K/mechanistic target of rapamycin (mTOR)) plays a role in the regulation of cell proliferation [57]. Additionally, hyperinsulinemia increases hepatic lipid accumulation and, consequently, leads to oxidative stress due to the increased beta-oxidation of free fatty acids (FFAs) and formation of reactive oxygen species (ROS) [58,59]. There is positive feedback between oxidative stress in mitochondria and endoplasmatic reticulum (ER) through ER stress due to increased calcium efflux and consequent mitochondrial and lysosomal permeabilization, further contributing to cell injury. ER stress can be considered an ultimate contribution to hepatic cell injury and carcinogenesis in NASH [60,61]. In contrast to insulin-mediated apoptosis inhibition, hepatic lipotoxicity activates proapoptotic cell signals [59]. Another recently discovered mechanism involves the association between lipolysis and autophagy, with conflicting evidence due to its double-natured, divergent role in NASH-associated HCC [62,63]. However, its role in energy metabolism via the PI3K/mTOR pathway strongly supports autophagy as a future candidate for therapeutic purposes.

Due to the complex interplay between the microenvironmental changes and the genetic and metabolic mechanisms in NASH-related HCC pathogenesis, future therapeutic strategies must involve additional and/or synergistic effects using drugs that target multiple pathways, some of which are currently under phase II or III clinical trials [64,65,66,67,68]. However, a balance between control of steatosis, chronic inflammation and fibrosis and an adequate polytherapy safety profile is mandatory.

### 3.3. The Role of Gut Microbiota

Gut microbiota could be associated with oncogenic pathways that promote HCC development [69,70]. Metabolites of commensal bacteria protect colonic mucosal cells, sustain proper gut barrier and suppress local colonic and adipose tissue inflammation [71,72]. Intestinal barrier disruption results in leaky gut with increased intestinal permeability with upregulated bacterial translocation and accumulation of lipopolysaccharide (LPS) with a chronic inflammatory state [69,71]. Studies in gut-sterilized and germ-free mice or mice treated with microbiota-associated molecular patterns (MAMPs) and bacterial metabolites provided evidence that gut microbiota and microbially activated pathways lead to HCC development [73]. NAFLD-induced cirrhosis is associated with increased levels of Bacteroides and Ruminococcaceae, and a reduced amount of Akkermansia and Bifidobacterium [74]. Dysbiosis and leaky gut are found in the early stages of CLD and accelerate inflammation, steatosis and fibrogenesis [75,76,77]. Portal levels of LPS increase in line with liver impairment, with the highest concentration in patients with Child–Pugh C cirrhosis [75]. Hepatic carcinogenesis in mice that was evoked by the combination of diethylnitrosamine (DEN) and carbon tetrachloride (CCl4) was associated with an inflammatory process that was mediated by LPS and toll-like receptor 4 (TLR4). In contrast, carcinogenesis that was activated by 7,12-dimethylbenz[a]anthracene (DMBA) and a high-fat diet was accompanied by inflammation that was mediated by lipoteichoic acid and TLR2 [78,79]. TLR4 mediates hepatocarcinogenesis via resident hepatic cells, such as hepatic stellate cells (HSCs), macrophages and hepatocytes. Additionally, TLR4 potentiates hepatic fibrosis and upregulates HSCs-derived hepatomitogen epiregulin [78]. Chronic low-dose LPS infusion evokes HCC development in mice, while TLR4 activation in mice with a lack of HSCs increases inflammatory gene expression and tumor cell proliferation. Dysbiosis and a high-fat diet cause the accumulation of Gram-positive bacteria with an enhanced capacity for the conversion of bile acids (BAs) [80]. TLR2, in collaboration with deoxycholic acid (DCA), which is a secondary BA, mediates carcinogenesis in a fatty liver by inducing a senescence-associated phenotype of HSCs, which, in turn, acquires profibrogenic and tumor-promoting abilities [80,81].

A high content of secondary BAs, such as DCA, influences chemokine ligand 16 (CXCL16) expression in sinusoidal endothelial cells (SECs). CXCL16 expression correlates positively with the primary BA, namely, chenodeoxycholic acid (CDCA), and negatively with DCA [82]. A commensal microbiota plays a role in the increase of CXCR6+ natural killer T (NKT) cells and heightened interferon production upon antigen stimulation. NKT cells mediate selective liver tumor inhibition. CXCL16 expression on SECs regulates NKT cell accumulation and is increased by the primary BA [83]. Secondary BAs are also capable of activating the mechanistic target of the rapamycin (mTOR) pathway in hepatocytes. A reduction of secondary BAs using antibiotic treatment was shown to inhibit NASH-derived HCC that was triggered by a high-cholesterol, high-fat NASH diet and mTOR activation [84]. Dysbiosis may also lead to increased production of trimethylamine (TMA) and trimethylamine N-oxide (TMAO) from absorbed dietary choline and carnitine. Decreased choline levels due to its conversion to TMA increase the risk of hepatotoxicity, whilst upregulated TMAO potentiates insulin resistance, potentially resulting in hepatocarcinogenesis [85]. Some experimental studies showed how both innate and adaptive immune responses influence hepatocarcinogenesis. However, less is known about the potential impact of gut microbiota on hepatic immunosurveillance [86].

Taken together, these data suggest that modulation of the gut microbiota may represent a new avenue to treat or prevent the development of liver injury and HCC in NAFLD.

### 3.4. Immune-Mediated Mechanisms

Hepatocyte damage and death are the central events that drive inflammation and HCC formation in NAFLD, although the exact process leading to cancer is not known. In NAFLD, hepatocyte death is the result of lipotoxicity resulting from inappropriate lipid and free fatty acid (FFA) accumulation in hepatocytes, which causes oxidative stress [87,88]. Damaged hepatocytes release damage-associated molecular patterns (DAMPs) that cause liver resident macrophages called Kupffer cells (KC) to activate and secrete pro-inflammatory cytokines, such as IL-1β and IL-18 [89,90]. In addition, NAFLD was associated with gut dysbiosis and impaired gut permeability [91,92,93]. Leaky gut permits pathogen-associated molecular patterns (PAMPs), such as bacterial lipopolysaccharide (LPS), to enter the portal circulation and lead to further KC activation [94]. These liver-specific macrophages display different types of receptors [95]. However, activation through pattern recognition receptors (PRR), such as toll-like receptors (TLRs), is responsible for the release of pro-inflammatory cytokines, such as tumor necrosis factor—α (TNF-α), IL-1, IL-6, CCL2 and CCL-5, that further perpetuate the influx of other immune cells, such as monocyte-derived macrophages, neutrophils and lymphocytes, to the liver, causing an adaptive Th17-mediated immune response and resulting in escalating and perpetuating chronic inflammation involving various cell lineages [96,97,98,99].

Evidence suggests that HCC occurs more frequently in NASH cirrhosis than in NASH without cirrhosis, highlighting the central role of fibrosis in cancer development [100,101]. It was shown that the expansion of the macrophage pool in the liver by infiltrating monocytes promotes steatohepatitis and fibrosis progression [97,102,103,104]. Hepatic stellate cells (HSCs) represent the major source of collagen fibers that constitute the extracellular matrix (ECM). Kupffer cells activate HSCs using different pathways. Bacterial LPS activates KC through TLR-4, causing them to produce transforming growth factor-β (TGF- β) and platelet-derived growth factor (PDGF), which, in turn, leads to the activation of HSCs and possibly HCC [78,99,105]. However, other receptors, such as TLR-9, may also play a role [106]. Hepatic progenitor cells (HPC) also seem to contribute to HCC by expressing profibrogenic factors [107]. Once activated, HSCs secrete various angiogenic and regenerative cytokines, of which, PDGF-C seems to play a major role, creating a microenvironment that is suitable for HCC growth [108,109]. Furthermore, the activation of CD8+ lymphocytes and natural killer (NK) cells promotes NASH and HCC progression [110]. Indeed, NK cells seem to prevent fibrosis formation, but as the disease progresses, their function is diminished, which promotes HCC formation [111].

Immune cells also play a role in hepatocarcinogenesis through processes that are independent of fibrosis [112]. Indeed, hepatocyte damage promotes neutrophil infiltration in the liver, resulting in DNA damage to other hepatocytes and promoting HCC development without fibrosis [113]. Furthermore, lymphoid aggregates that consist of infiltrating lymphocytes are often present in the setting of chronic inflammation. Through chronic NF-κB activation, these structures also promote HCC development [114]. Other examples include the selective loss of CD4+ T lymphocytes, which was shown in mouse models to be critical for the progression of HCC [115]. This observation was further confirmed in HCC patients in whom CD4+ lymphocyte loss was correlated with poor survival and high recurrence rates [116].

Although the immunological response can promote cancer formation, the immune system also plays an important role in suppressing tumor growth through immunosurveillance. Severe defects in immunosurveillance were observed in mouse models that were fed with a high-fat, high-sugar diet (HFHSD) [117]. Furthermore, HCC actively promotes tumor tolerance by inducing immunosuppression, and the fibrotic microenvironment leads to the overproduction of TGF-β, which is a potent immunosuppressant, thereby promoting disease progression [117,118].

## 4. Clinical Picture and Outcomes

### 4.1. Clinical Presentation of HCC in NAFLD

The clinical presentation of NAFLD is usually silent regarding the liver, as the symptomology is often dominated by the accompanying metabolic conditions, such as obesity, diabetes and arterial hypertension [1].

Other than a dull ache, or rarely pain in the right upper abdominal quadrant, as well as fatigue, most patients with NAFLD do not report symptoms that might point to a diagnosis of liver disease [1]. The development of liver decompensation may be the first sign of liver disease, and HCC may be diagnosed based on symptoms of cancer cachexia, or tumor-related complications, such as tumor rupture, bleeding, biliary obstruction, pain or portal hypertensive complications caused by portal vein infiltration. From a clinical perspective, the most favorable scenario is when HCC is found using imaging studies that are performed during surveillance or as an incidental finding during investigation for another condition.

Although surveillance for patients with liver cirrhosis has been endorsed by all relevant hepatological societies around the world, the real-life results in terms of the stage at which the majority of HCC are detected are suboptimal [5,6,119]. According to the BRIDGE study, HCC is discovered with Barcelona Clinic Liver Cancer (BCLC) stage C in most cases (>50%) in North America, Europe, China and South Korea, whereas in Taiwan and Japan, most patients (around 70%) are diagnosed with stage 0-A, probably as the result of more rigorous screening programs [19].

More specific data about the patterns of HCC clinical presentation in NAFLD come from a multicentric Italian study that included 145 patients with HCC and NAFLD and 611 patients with HCC and chronic hepatitis C [120]. Patients with NAFLD-related HCC were younger (mean 67.8 vs. 71.1 years), more often males (79.3 vs. 61.2%), diagnosed at more advanced stages (BCLC stage > A in 50.6 vs. 43.4%), revealed more infiltrative pattern (21 vs. 4%) and almost 50% occurred in non-cirrhotic liver. Patients with NAFLD were also less frequently diagnosed as part of a surveillance protocol (47.6 vs. 63.3%) and had significantly lower serum levels of alpha-fetoprotein (AFP) (median 7.13 vs. 20.4 ng/dL).

Patients presenting with NAFLD-related HCC are frequently burdened with multiple comorbidities, especially diabetes [24]. Indeed, studies from the USA reported a 2.8-fold higher risk of developing HCC among NAFLD patients who had diabetes, rising to over fourfold in patients with the concomitant presence of diabetes and NAFLD cirrhosis [24,27]. As already discussed, cirrhosis is present at the time of HCC diagnosis in the majority of patients but may be absent in around 30% of cases [24,27,121].

Amongst Japanese patients with NAFLD-related HCC, advanced fibrosis and cirrhosis were present in 72% of patients at the time of diagnosis, and more than 50% of patients were obese or had diabetes or arterial hypertension [121]. Men were more likely to develop HCC in a non-cirrhotic liver.

Therefore, NAFLD-related HCC is usually diagnosed in patients with advanced fibrosis and cirrhosis but can occur in the absence of advanced fibrosis/cirrhosis in around 20–50% of cases. It is associated with the presence of metabolic syndrome, with the strongest relative influence from diabetes. NAFLD patients that are affected by HCC are mostly males, in their sixties, less frequently diagnosed during the surveillance protocols and with more advanced clinical stage and infiltrative tumor pattern, preserved liver function and low AFP serum level.

### 4.2. Clinical Outcomes of Patients with NAFLD-Related HCC

The average 5-year survival of HCC patients is generally poor, ranging from 5 to 14%, depending on several factors, including the tumor stage at diagnosis, liver functional status, patient’s performance status and the availability of adequate medical care [122,123,124]. According to the results from the BRIDGE study, the overall survival (OS) according to BCLC stage was 80, 27, 15 and 4 months for the A, B, C and D stages, respectively [19]. As most patients were diagnosed with BCLC stage A, the median overall survival was not reached in Taiwan, whereas it was 60 months in Japan, as opposed to a median OS of 33, 31, 24 and 23 months in North America, South Korea, Europe and China, respectively where the most common BCLC stage at diagnosis was C. These data clearly underscore the importance of surveillance, as both countries with already implemented National screening programs (Taiwan and Japan) demonstrated favorable results in terms of the early detection of HCC, resulting in increased eligibility for potentially curative treatments, resulting in an increased OS.

As for the NAFLD-related HCC, the outcomes are in general inferior to HCV-related HCC due to the more advanced stage at diagnosis (survival rates at 1 and 3 years were 76.4 and 48.7% vs. 84.2 and 61.1%, respectively) [120]. However, when adjusted for covariates (age, HCC stage, type of treatment), these differences disappear and the outcomes are the same. To illustrate this, patients referred for curative treatments (within Milan criteria) had mean survivals of 38.6 months (NAFLD) and 41 months (HCV), with these being statistically non-significant.

## 5. Diagnostic Approach

Current guidelines recommend US surveillance every 6 months for NAFLD patients with advanced fibrosis or cirrhosis [5,6,119]. However, there is much less certainty about the diagnostic approach to patients in the absence of advanced fibrosis or cirrhosis. The most important issue is probably the awareness of the presence of the risk factors for chronic liver disease. Diabetes mellitus is the strongest individual risk factor for the development of cirrhosis and HCC, with further increased risk from other coexisting metabolic traits [24]. These patients may also exhibit harmful alcohol drinking habits, which is concerning since alcohol is an additional risk factor for the development of cirrhosis and HCC. Ideally, NAFLD patients should undergo screening for the presence of advanced fibrosis/cirrhosis using non-invasive methods to stratify for the presence of advanced fibrosis or cirrhosis. Currently, about 60% of the European population is considered at risk of having chronic liver disease, and many studies are underway to develop the most suitable diagnostic algorithm to reliably recognize patients who are at risk for advanced liver fibrosis [125,126]. Stepwise or even concomitant use of two unrelated non-invasive tests might be the most favorable strategy to increase the reliability of the obtained result, i.e., predicted stage of liver fibrosis, as supported by guidelines [16,124,125,126,127,128]. However, even transient elastography (TE) as the non-invasive method considered among the most reliable in stratifying the patients according to their risk of having advanced fibrosis, as supported by the largest body of scientific evidence, is not without limitations [129]. Indeed, according to the published data, this method tends to overestimate the presence of advanced fibrosis amongst patients with NAFLD, as the stage suggested by TE was confirmed by liver biopsy in only 50% of patients in a cohort of patients with type 2 diabetes [130]. Therefore, the diagnosis of advanced fibrosis/cirrhosis should rely on concordant results of two unrelated non-invasive tests (elastographic and serological) and may still need confirmation using a liver biopsy, as many NAFLD patients also drink alcohol, which, together with steatosis, might influence the estimated stage of liver fibrosis using elastography. If advanced fibrosis is confirmed, this would qualify the patients for the further step, i.e., inclusion in surveillance programs.

Another possible approach is to perform a US on each patient that is suspected to have NAFLD [131]. This approach might potentially increase the number of HCC that is detected in the early stage, but as was already discussed, does not meet the criteria for cost-effectiveness and, due to the numbers involved, is not feasible. To illustrate this here, we simulate the possible situation in Croatia, which is a small central-eastern European country with a population of around 4 million. Provided that 25% of the population has NAFLD, this would mean that 1 million should undergo an index US examination. According to the data from an audit that was conducted by the US section of the Croatian Society of Gastroenterology during the first 6 months of 2018, the number of US examinations (abdominal USs) that were performed by all Croatian gastroenterologists working in the public sector was 32,000, and about 27% of patients were referred for liver-related indications (personal data of the corresponding author). Therefore, to screen 1 million people by performing 64.000 USs a year (only for the purpose to screen patients under suspicion of having NAFLD, and no other indications) it would take at least 15 years to perform the index USs (not to mention surveillance in patients with cirrhosis or repeated examinations in doubtful cases). Even if the target population is narrowed to only patients with type 2 diabetes, assuming the prevalence of 9–10%, giving the total number of around 400,000 patients, it would take 5 years to perform the index abdominal USs for these patients only (without doing USs for any other indication), which is currently unrealistic.

Ultrasound is currently recommended for the surveillance of NAFLD patients with advanced fibrosis or cirrhosis [5,6,119,132]. It has a sensitivity of only 47% to detect early-stage HCC [133]. In addition to this, 20% of US examinations for patients with cirrhosis are of inadequate quality to rule out the presence of HCC, mostly due to US artifacts, inadequate US penetration and patient-related characteristics, such as obesity, Child B/C cirrhosis and alcohol or NASH related cirrhosis. Furthermore, there are some areas of the liver that are unreliable for US examination, such as the subcapsular regions [134]. In this context, another important issue is the quality of the US equipment and the experience of the ultrasonographer [133]. The detection rate of HCC has not changed substantially over the two decades from the late 1980s until now according to the report from the Italian multicentric study that encompassed 1170 HCC patients, arguing against the importance of the location at which the US examination was delivered (primary or tertiary care center), as well as against the decisive role of the type and level of the US equipment [135]. Nevertheless, data from Canada reflecting a more recent period demonstrated that the number of accurately diagnosed HCC using US surveillance increased from 2000 to 2010, potentially as the result of better US equipment [136].

US may also overdiagnose the presence of HCC in cases of macronodular cirrhosis and in patients with very irregular and coarse liver parenchyma [132]. This may be harmful, as it often calls for additional investigations, such as repeated computerized tomography (CT) examinations, MRI and sometimes even a liver biopsy [137].

Biomarkers might represent an attractive alternative or additional tool for the early detection of HCC [124,137]. Whilst AFP remains the only biomarker for surveillance purposes that is currently endorsed by international guidelines, its diagnostic performance is moderate and new candidates have recently been proposed, such as serum inter-alpha-trypsin inhibitor heavy chain 4 (ITIH4) and the GALAD score [138,139,140]. ITIH4 was identified using proteomic analysis as the most prominent protein that is associated with NAFLD progression, including the development of HCC using pigs that were fed a hypercaloric diet [138]. When validated in humans, patients with NAFLD-related HCC had a significantly higher serum level of ITIH4 compared to those with simple liver steatosis and NASH. The GALAD score combines demographic parameters, such as age and sex, with the serum AFP, AFP-L3 and des-gamma-carboxy-prothrombin (DCP), and was reported to have significantly better diagnostic performance in comparison with AFP (AUROCs 0.96 vs. 0.88, respectively, *p* < 0.0005) [139]. The best performance for the detection of early HCC was achieved at the cut-off level of -1.134 (sensitivity 86.21%, specificity 90.91%, positive predictive value 0.54, negative predictive value 0.98 and 90.4% of cases were correctly classified). GALAD score had similar performance both for patients with and without NASH cirrhosis (AUROC 0.93 and 0.98, respectively). Interestingly, the GALAD score was elevated as early as 1.5 years beforehand in patients with NASH who were subsequently diagnosed with HCC. Finally, the combination of GALAD score with US (GALADUS score) was demonstrated to improve the diagnostic performance of both methods (AUROCs for US alone, GALAD score and GALADUS score were 0.82, 0.95 and 0.98, respectively, for any size of HCC) [141]. For early HCC (BCLC 0-A), the respective AUROCs for the GALAD and GALADUS scores were 0.92 and 0.97. These data were retrieved from a cohort of patients with mixed etiology of CLD and need further validation, specifically for patients with NAFLD.

**Figure 2:**
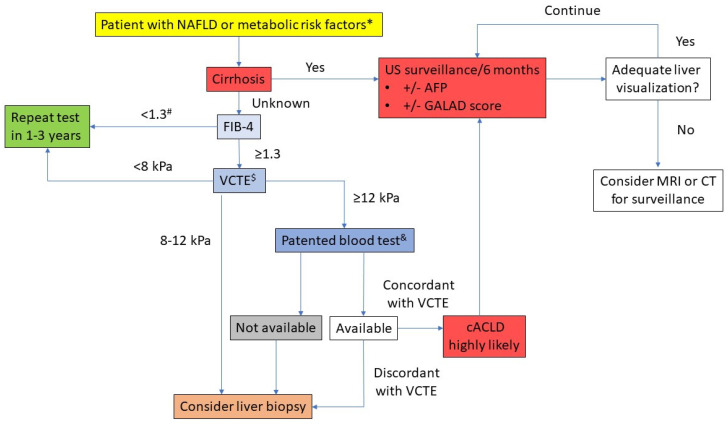
Proposed diagnostic algorithm for stratifying the risk of hepatocellular carcinoma and surveillance in patients with NAFLD. FIB-4—fibrosis-4 index; VCTE—vibration-controlled transient elastography; cACLD—compensated advanced chronic liver disease; AFP—alpha-fetoprotein; MRI—magnetic resonance imaging; CT—computerized tomography. * Presence of type 2 diabetes mellitus or any two of the following: central obesity, elevated triglycerides or low HDL cholesterol, arterial hypertension and insulin resistance. ^#^ FIB-4 < 2.0 in patients >65 years. ^&^ Other simple and validated non-invasive tests based on routine blood parameters may be used instead. ^$^ Other ultrasound-based elastography methods may be used, but they have less well-defined cut-offs. Cut-offs: Enhanced Liver Fibrosis (ELF™ test) 9.8; Fiobrotest 0.48; FibroMeter 0.45. Adapted based on references [127,128,142,143,144].

The diagnostic algorithm for stratifying the risk of HCC and surveillance in patients with NAFLD is proposed in Figure 2.

## 6. Conclusions

In conclusion, NAFLD is becoming the leading cause of liver morbidity worldwide, and, as such, represents the pathogenic background for the increasing incidence of HCC. The annual incidence of NAFLD-related HCC is expected to increase by 45–130% by the end of this decade. Diabetes mellitus is the most important risk factor for HCC development in NAFLD, with further increases in risk when it is associated with other co-morbidities, such as obesity, arterial hypertension and dyslipidemia. The highest risk of HCC exists in patients with advanced fibrosis or cirrhosis, although 20–50% of HCC cases arise in NAFLD patients in the absence of cirrhosis. This calls for further investigation of pathogenetic mechanisms that are involved in hepatocarcinogenesis, including the genetic background, metabolic disturbances, influence of gut microbiome and immunological response. Apart from primary prevention, one of the most important goals to improve the survival of these patients is the early recognition of the presence of NAFLD based on easily assessed demographic and clinical characteristics. Patients should then be stratified for the presence of advanced cirrhosis or fibrosis and, subsequently, enter HCC surveillance programs. The early recognition of non-cirrhotic NAFLD patients who will develop HCC remains a significant challenge, and new candidate biomarkers and scores are likely to be candidates to tackle this issue.

## Figures and Tables

**Table 1:** Rates of hepatocellular carcinoma (HCC) attributable to non-alcoholic fatty liver disease (NAFLD) and those arising in non-cirrhotic liver, according to the geographic region and period.

HCC Cases Attributable to NAFLD				
Region	Investigated Population	Year/Period	Prevalence (%)	Reference
North America Europe Asia–Pacific Africa	Multicentric study with 42 sites included in 14 countries across the world involving 18,031 patients diagnosed with HCC.	2012–2015	12 10 1–6 12–22	[20]
United States	4406 HCC patients identified within a healthcare claims database covering 18 million lives yearly and all US census regions.	2002–2008	58	[19]
United States	26,121 transplanted or waitlisted patients with HCC identified from the Scientific Registry of Transplant Recipients.	2002 2017	2.1 16.2	[21]
United Kingdom	632 HCC patients consecutively presented at the multidisciplinary team covering North East England, Cumbria and North Yorkshire.	2000 2010	<8 34.8	
France	323 consecutive patients who underwent liver resection due to HCC at two tertiary centers in Paris over a 20-year period.	1995–1999 2010-2014	2.6 19.5	[22] [23]
**HCC Arising in the Absence of Cirrhosis**				
Germany United States Japan Germany South Korea	Systematic review with meta-analysis: 19 studies with 168 571 participants and available data about the presence of HCC among patients with/without cirrhosis; 13,345 patients had NAFLD; overall prevalence of HCC in non-cirrhotic NAFLD was 38%.	2007–2008 2000–2010 2006–2009 1994–2013 2005–2012	41.7 26.9 38 13.9 34.3	[24]

**Table 2:** Overview of the genetic mechanisms in non-alcoholic fatty liver disease (NAFLD)-related hepatocellular carcinoma (HCC) pathogenesis. Abbreviations: *CDKN2A*—cyclin-dependent kinase inhibitor 2A; *CHD1*—chromodomain-helicase DNA-binding 1; *CTNNB1*—catenin beta 1; DM—diabetes mellitus; *HFE*—hemochromatosis gene; *MET*—methionine; miRNAs—microRNAs; NAFLD—non-alcoholic fatty liver disease; NASH—non-alcoholic steatohepatitis; *PNPLA3*—patatin-like phospholipase domain-containing protein 3; *PTEN*—phosphatase and tensin homolog deleted on chromosome 10; *TERT*—telomerase reverse transcriptase; *TM6SF2*—transmembrane 6 superfamily member 2; TP53—tumor protein 53. (Adapted based on the references [31,32,33,34,35,36,37,38,39,40,41,42,43,44,45,46,47,48,49,50,51,52,53]).

Mechanisms		Role in Pathogenesis
Single Gene Mutation	*PNPLA3, rs738409, p.Ile148Met, chr22*	Increased liver lipid accumulation with a predisposition toward fatty hepatic diseases (NAFLD, NASH, HCC). Influences liver storage of retinol in obese patients, with role in HCC stellate cells to be investigated.
	*TM6SF2*, rs58542926, p.E167K, chr19p13.11	Influences steatosis and advanced fibrosis, independently of DM, obesity or *PNPLA3* genotype. Shown in hepatic injury in NAFLD-related HCC, without a clear role in HCC progression.
	*HFE H63D*, *rs1799945, chr6p21.3*	Found in non-cirrhotic HCC and led to hepatic inflammation, fibrosis and carcinogensis due to increased parenchymal iron accumulation.
	*rs641738, chr19q13.42*	Severe liver damage and increased fibrosis risk in NAFLD, with a further investigation regarding HCC progression.
Genetic Instability	DNA amplification of genes involved in oncogenic mechanisms (*TERT, VGFA, MET, MYC*)	Inducements for NAFLD-related HCC.
	Oncogene mutations (*CTNNB1, AXIN1, ALB, TP53, CDKN2A*)	
	*XPO4 and PDE1B* genes	Identified in NAFLD-related HCC; unknown physiological roles in NAFLD-related HCC.
Epigenetic Changes	*CHD1* gene	DNA methylation leading to gene silencing; related to DNA damage and repair, lipid and glucose metabolism and fibrosis progression.
Dysregulated microRNA Expression	Downregulated liver-specific *miR-122*	Reduced expression in NAFLD with negatively regulated hepatic lipogenesis.
	Other miRNAs’ altered expression (*miR-21, miR-29, miR-23, miR-155, miR-221, miR-222, miR-106, miR-93, miR-519*)	Major hepatocarcinogenic pathways with several targeting the PTEN protein.
